# Response to Letter to the Editor from Maureen McCormick: Obesity and Inflammation: Change in Adiponectin, C-Reactive Protein, Tumour Necrosis Factor-Alpha and Interleukin-6 After Bariatric Surgery

**DOI:** 10.1007/s11695-012-0788-8

**Published:** 2012-10-18

**Authors:** Fátima Illán Gomez

**Affiliations:** Department of Endocrinology and Nutrition, University Hospital Morales Meseguer, C/ Marques de los Velez s/n, 30008 Murcia, Spain

Dear Dr. Maureen McCormick,

We have carefully read with great interest your comments and concerns about the statistical results of our study. We would like to make some remarks.

If we repeat the statistical analysis of our data using an analysis of variance for repeated measures, we will obtain statistically significant results similar to our previous results, both globally or in pairs except for basal and 3-month interleukin-6 (IL-6) levels (*p* = 0.3).

We have used a parametric analysis because our sample is composed of more than 30 patients, wherein this kind of test is appropriate. However, if we use the Friedman non-parametrical test for our data, we will still obtain statistically significant results similar to the results that we have described in our article. There was a globally marked statistically significant reduction for the whole duration of the study (*p* < 0.001) in the levels of IL-6, adiponectin and C-reactive protein. We have also found statistical differences for tumour necrosis factor-alpha but with the variations described in the study.

We appreciate sincerely your point of view. We are sure that you will help us to improve the quality of our article.

Best regards,

Fátima Illán Gomez

